# The role of metal ion metabolism in the pathogenesis of diabetes and associated complications

**DOI:** 10.3389/fendo.2025.1541809

**Published:** 2025-04-03

**Authors:** Siyuan Liu, Xuzhuo Chen, Xinrui Qi, Jiahao Bai, Bin Tong, Deju Zhang, Xiaoping Yin, Peng Yu

**Affiliations:** ^1^ Jiujiang Clinical Precision Medicine Research Center, Jiujiang, Jiangxi, China; Department of Endocrinology and Metabolism, the Second Affiliated Hospital, Jiangxi Medical College. Nanchang University, Nanchang, Jiangxi, China; The Second Clinical Medical College of Nanchang University, Nanchang, Jiangxi, China; ^2^ Jiujiang Clinical Precision Medicine Research Center, Jiujiang, Jiangxi, China; Department of Endocrinology and Metabolism, the Second Affiliated Hospital, Jiangxi Medical College, Nanchang University, Nanchang, Jiangxi, China; ^3^ Laboratory of Pharmacy and Chemistry, Lab Teaching & Management Center, Chongqing Medical University, Chongqing, China; ^4^ Food and Nutritional Sciences, School of Biological Sciences, The University of Hong Kong, Hong Kong, Hong Kong SAR, China; ^5^ Department of Neurology, Affiliated Hospital of Jiujiang University, Jiujiang, China; Center for Clinical Precision Medicine, Jiujiang University, Jiujiang, China

**Keywords:** diabetes, metal ion, metabolism, iron ion, copper ion, complications

## Abstract

Diabetes is a growing health concern, accompanied by significant complications like cardiovascular disease, kidney disease, and retinopathy. Metal ions, including iron, zinc, and copper, play a crucial role in maintaining human health through their balance within the body. Disruptions in metal ion balance can intensify diabetic conditions. For instance, iron overload induces oxidative stress, which harms islet β cells and impacts vascular complications of diabetes. Abnormal copper levels heighten insulin resistance, and zinc deficiency has a strong connection with type 1 diabetes. Future in - depth exploration of the association between metal metabolism and diabetes holds the potential to uncover novel treatment avenues, enhancing both the quality of life and health prognosis for patients.

## 1 Introduction

The increasing global prevalence of obesity and unhealthy lifestyles has contributed to the rising incidence and prevalence of diabetes-related diseases. The International Diabetes Federation (IDF) reported that, in 2021, 10.5% of adults aged 20 to 79 had diabetes. Among all diabetic individuals, type 1 diabetes mellitus (T1DM) accounts for between 5-15%, and type 2 diabetes accounts for the majority. It is currently estimated that the number of people living with diabetes will increase from 536.6 million to 783.2 million by 2045 (1).

Diabetes can be divided into type 1 diabetes mellitus (T1DM) and type 2 diabetes mellitus (T2DM). The main difference between the two is the different inactivation pathways of pancreatic β cells. T1DM is an autoimmune destruction of beta cells, which usually leads to insulin deficiency, while T2DM is a frequent progressive decrease in beta cell insulin secretion and insulin resistance (2). Based on current research, metal elements play a crucial role in the human body, and several essential metals are required for many enzymes, transcription factors, and proteins to play important roles in various biochemical pathways. In diabetes, metal ions play an indispensable role. For example, chromium can enhance the action of insulin and help lower blood glucose levels (3). Zinc is involved in the synthesis and release of insulin and affects blood glucose regulation (4). Copper plays a role in redox reactions and may affect insulin function (5). The imbalance of these metal ions may lead to the development or aggravation of diabetes. Additionally, the plasma levels of trace metal elements such as magnesium, copper, selenium, and zinc differ between patients with T1DM and T2DM. In T1DM, the plasma magnesium concentration changes most and decreases. Besides, in T2DM, plasma levels of selenium and copper were significantly affected, and the plasma selenium and copper concentrations decreased (6).

Diabetes is associated with high rates of microvascular (neuropathy, nephropathy, and retinopathy) and macrovascular (coronary artery disease, cerebrovascular disease, and peripheral artery disease) complications. There is a close relationship between metal ions and diabetic complications. For example, chromium levels may decrease in hyperglycemic states, which may affect insulin sensitivity and increase cardiovascular disease risk (7). Zinc deficiency may lead to impaired insulin synthesis and secretion, affecting glycemic control (8). The role of copper in diabetic complications is still under investigation, but its involvement in REDOX reactions may affect vascular health and neurological function (9).

Investigating the connection between diabetic complications and metal ions could help us to understand the pathogenesis and offer new targets and strategies for treatment and prevention, thereby enhancing patients’ quality of life and decreasing the complication incidence.

## 2 Definition of metal ion metabolism

### 2.1 Types and functions of essential metal ions in the human body

Essential metals in the human body include Na, K, Ca, Mg, Zn, Cu, Fe, Mn, Co, and Mo (10). Metal elements play a vital role in the human body (11) (**Table 1**). For example, iron ions contribute to the construction of hemoglobin and participate in oxygen transport in the body. Copper ions serve as cofactors for various enzymes, participate in iron transport, and promote angiogenesis. Zinc ions are components of zinc-containing metalloenzymes and zinc finger proteins, and are essential for insulin synthesis. Calcium ions are involved in building and maintaining bone health, maintaining neuromuscular excitability, and regulating the activity of many enzymes.

**Table 1:** Metalloprotein species of essential metals and their functions.

Essential metals	Metalloproteins	Function
Fe	Hemoglobin	Oxygen carrying and storage
	Myoglobin	Oxygen carrying and storage
	Cytochrome C	Electron transporter
Cu	Cu, Zn superoxide dismutase (SOD1)	Superoxide detoxification, signaling
	Lysyl oxidase (LOX)	Crosslinking of collagen and elastin
	Ceruloplasmin (Cp)	Ferroxidase
	Cytochrome c oxidase	Oxidative phosphorylation
Zn	Carbonic anhydrase	Catalyze the hydration of carbon dioxide
	Zinc finger protein	Bind nucleic acids (DNA or RNA)
Ca	Calcium-binding protein	Control cytoplasmic Ca^2+^ concentration. Function as Ca^2+^ transporters and calcium-sensors with regulatory potential

### 2.2 Definition of the regulation of metal ion metabolism

**Figure 1:**
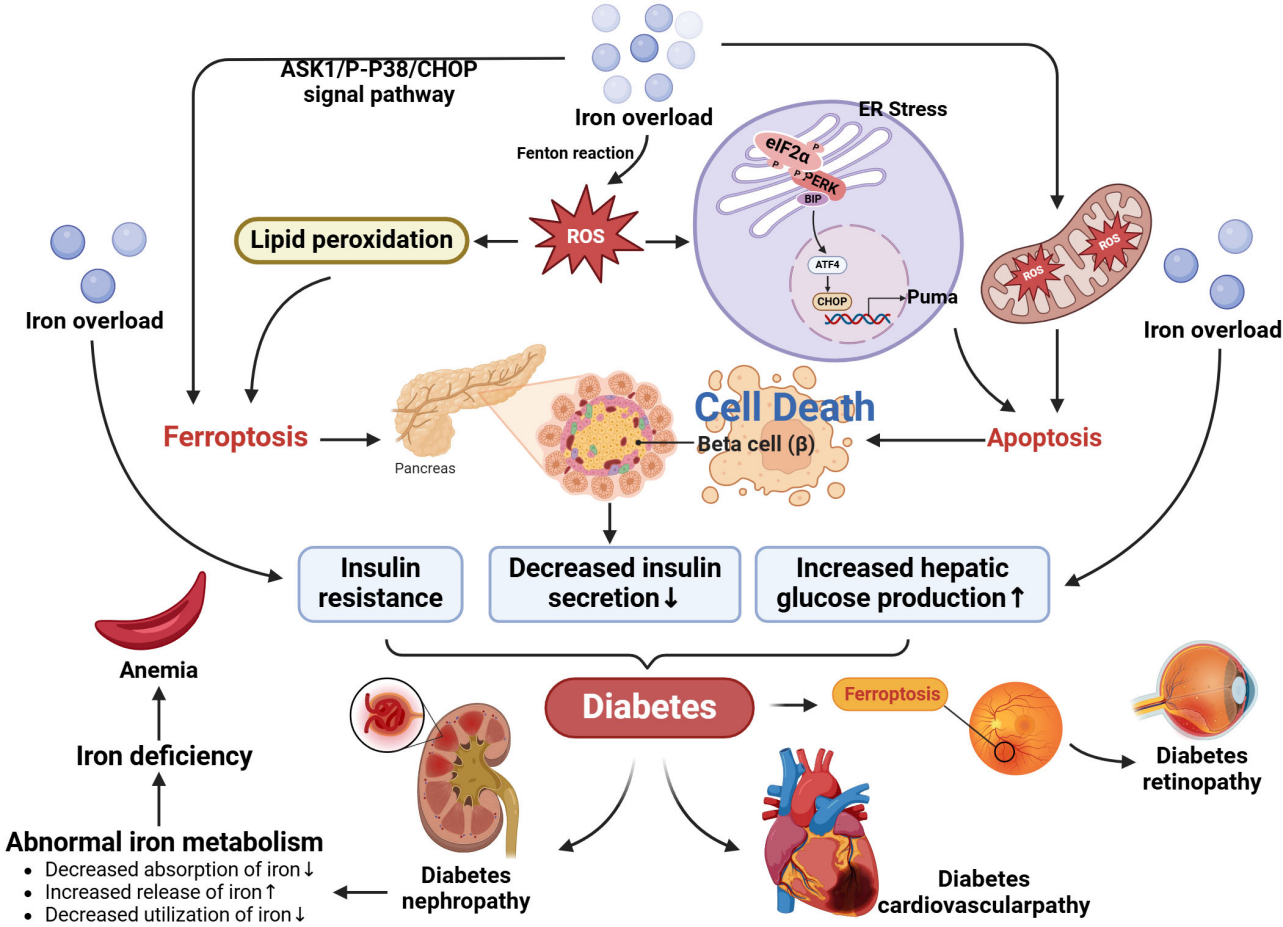
Metal ion metabolism. The main components involved in metal ion metabolism and homeostatic regulation include transporters, metal-blinding chaperones, and metalloproteins.

Metal ion metabolism refers to the regulation of the absorption, distribution, storage, and removal of metal ions in cells (12). Intracellular metal ion balance refers to the maintenance process of intracellular metal ion levels within a certain range. The steady-state regulation of metal ions is mainly through accurate absorption and adaptation (such as Zn, Fe, Cu, and Mn). Studies have shown that a critical absorption pathway is mediated by transporters located at the brush border of the intestine, which can selectively collect metal ions. The homeostatic regulation of absorption appears to depend on the expression of these proteins and/or molecules in the following transport line from the cell lumen to the bloodstream. However, renal excretion is less adaptive (Zn), and usually cannot quantitatively promote metabolic regulation (Fe, Cu, Mn) (13). For example, the regulation of iron ion homeostasis, which regulates the binding and dissociation of iron-regulated sequences (IREs) and iron-regulated proteins (IRPs) at the protein synthesis level to regulate the production of ferritin, which in turn maintains iron ion homeostasis (14). During the absorption stage, iron ion homeostasis is also regulated by the intestinal mucosa. Ferritin is an essential mechanism for regulating iron homeostasis in absorbing epithelial cells. When iron overload occurs, newly formed epithelial cells contain ferritin, which binds iron to ferritin. Iron is lost when cells are shed at the end of their life cycle. The iron that bypasses ferritin will be intercepted by macrophages in the villi. When iron is lacking, the absorption cells will lack ferritin, and the entry of dietary iron into the body is relatively unimpeded (15). The precise regulation of copper ions is achieved by metal chaperones. (see 4.1) (the mechanism is shown in **Figure 1**).

In addition, the absorbed metal ions will bind to the transporter and be transported to the target site. For example, iron is exported to the circulation through the basolateral membrane of intestinal epithelial cells, bound to transferrin and transported to the sites of utilization and storage. The iron bound with transferrin enters the target cells, primarily red blood cells, through receptor-mediated endocytosis, and iron is stored in hemoglobin and myoglobin (14). At the same time, copper is bound to albumin after intestinal absorption and transported to the liver, and then released into the blood and other tissues. Metallothionein is bound to copper in the liver (16).

### 2.3 Harm of metal ion metabolism disorder

If metal ion metabolism is out of balance, it can have a variety of adverse effects on human health, including:

Iron deficiency leads to iron-deficiency anemia, while iron overload can lead to iron deposition in organs such as the heart, liver, and brain, increasing the risk of diabetes, cirrhosis, liver cancer, arrhythmia, heart failure, retinal degeneration, and neurodegenerative diseases (17). Copper deficiency leads to anemia, neutropenia, osteoporosis, and neurological problems, while copper overload leads to gastrointestinal symptoms, cirrhosis, and neurological diseases (18). Zinc deficiency can lead to growth retardation, glossitis, slow wound healing, hair loss, and decreased immunity (19). Calcium deficiency can lead to rickets, osteomalacia, hypertension, atherosclerosis, diabetes, neurodegenerative diseases, degenerative joint disease, etc. Calcium overload can lead to hypercalcemia and urinary calculi (20).

## 3 Iron ion metabolism

### 3.1 Relationship between iron overload and diabetes

Iron overload (IO) is a hazard factor for diabetes. In the pathogenesis of diabetes, iron has a direct and causal role, which is mediated through β-cell failure and insulin resistance (21). It impacts most of its essential features: decreased insulin resistance, insulin secretion, and increased hepatic glucose production (22). The liver maintains iron homeostasis through heparin (23). Heparin is involved in the storage, transport and release of iron ions in the body by regulating the expression of ferritin and transporter proteins in the liver (24). In addition, heparin also regulates the absorption of iron in the intestine and the recycling of iron in the liver to maintain iron balance in the body (25). According to clinical trials, patients with T2DM have reduced heparin levels and increased plasma iron and ferritin levels (26). On the other hand, recent research has revealed that the accumulation of lipid peroxides and free iron will induce a type of cell death called ferroptosis (27). The mechanism is that free iron catalyzes the Fenton reaction, induces cellular oxidative stress, generates a large quantity of free radicals, and triggers a sequence of cell damage reactions (28). Free radicals will attack beta cells, leading to impaired blood glucose regulation in the human body, and then develop diabetes. According to recent studies, CHOP is one of the ferroptosis inducers related to the activation of the PERK-eIF2α-ATF4 pathway that mediates endoplasmic reticulum stress (29). Iron overload may activate the ASK1/P-P38/CHOP/COX-2 pathway, thereby inducing severe oxidative stress, resulting in the accumulation of numerous lipid peroxides and the imbalance of REDOX status in cells, which in turn induces ferroptosis of β cells (30). Secondly, iron accumulates in hepatocytes and leads to insulin resistance through oxidative stress (31, 32). Its mechanism mainly involves two aspects: oxidative stress and lipid metabolism disorder. Oxidative stress interferes with the structure and function of the insulin receptor and reduces the activity of the insulin receptor, thereby weakening the action of insulin (33). In addition, oxidative stress may also damage the intracellular molecules of the insulin signal transduction pathway, such as insulin receptor substrate, insulin receptor substrate binding protein, insulin receptor kinase, and so on, leading to the blockage of insulin signal transmission (22). Oxidative stress is one of the essential mechanisms of insulin resistance by damaging all aspects of insulin signal transduction pathway. Excess iron ions can also lead to disorders of lipid metabolism, including lipid peroxidation, fat accumulation, and abnormal lipid metabolism (34).

### 3.2 Oxidative stress induced by iron overload damages islet beta cells

Iron causes damage to cells through producing reactive oxygen species (ROS) and free radicals from peroxides. Iron is a critical participant in redox reactions, so excessive iron ions can promote the progress of redox reactions, leading to the production of large amounts of ROS. Secondly, excessive iron ions may undergo a Fenton reaction with oxidizing substances such as hydrogen peroxide in the cell to produce more active oxygen radicals, such as peroxyl radicals and hydroxyl radicals (35). These ROS are able to oxidize cell membrane lipids, proteins, and nucleic acids, triggering oxidative stress responses and damaging cell structure and function (36). In addition, excessive iron ions may also affect mitochondrial function, interfere with the normal operation of the electron transport chain, and increase the production of ROS within mitochondria. Islet beta cells are particularly sensitive to ROS (22). Since the expression rate of antioxidants such as SOD2 and catalase is low, experimental data show that antioxidant enzymes are usually ±50% in the liver, while gene expression is significantly reduced in pancreatic islets. In the liver, the gene expression levels of mitochondrial Mn-SOD and cytoplasmic Cu, Zn superoxide dismutase (Cu, Zn-SOD) were in the range of 30 - 40%. In islets, the gene expression of glutathione peroxidase was 15%, while catalase gene expression was not detectable (37, 38).

### 3.3 Iron metabolism disorders and diabetic complications

**Figure 2:**
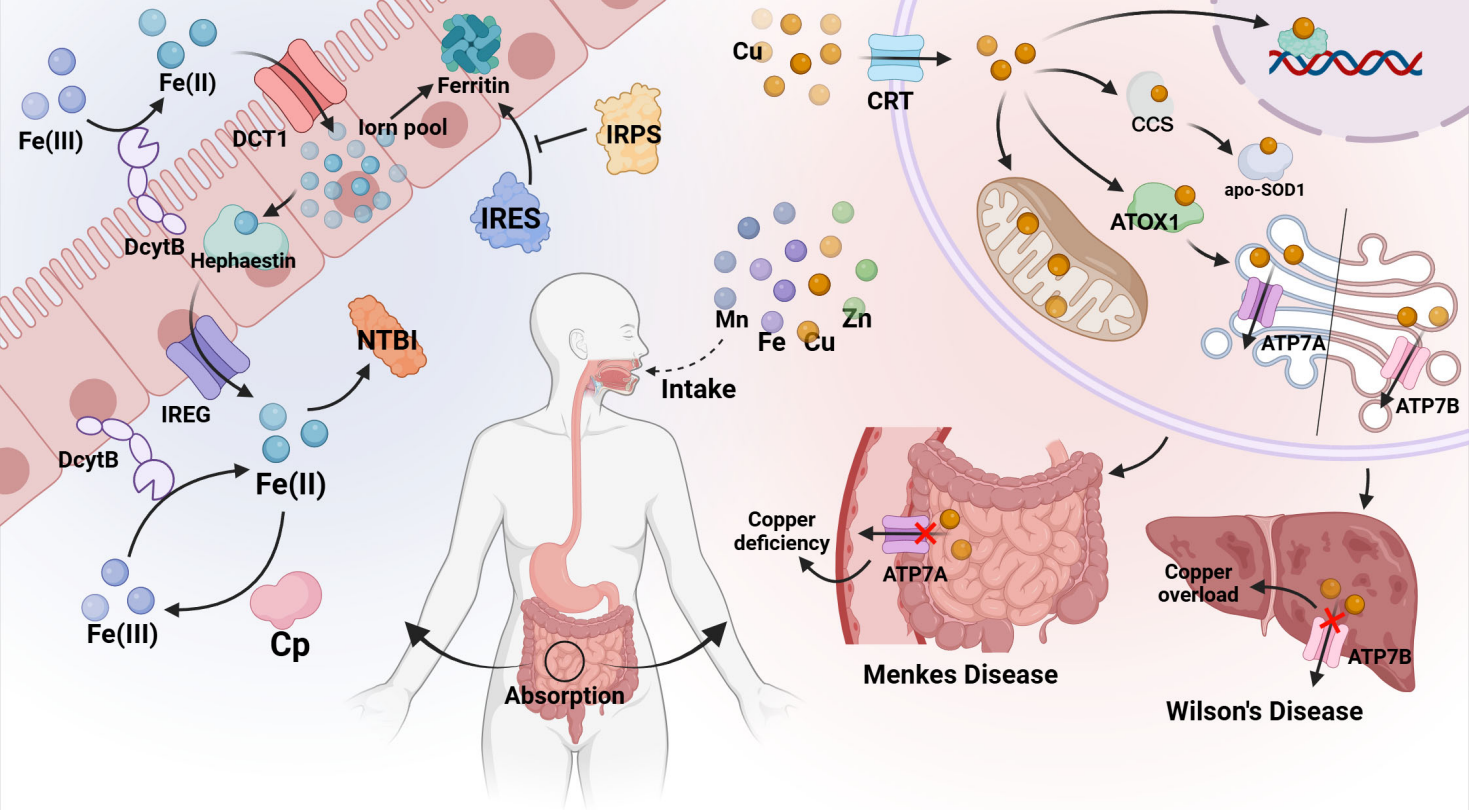
Relationship between iron metabolism disorder and diabetes and its complications. Plasma iron and ferritin levels increase in T2DM patients. Iron accumulates in liver cells and leads to insulin resistance and reduced secretion through oxidative stress pathways. In addition, free iron can induce iron death of beta cells by activating the ASK1/P-P38/CHOP signaling pathway. Besides, an iron influx in the retina, through the Fenton/Haber-Weiss reaction, causes the generation of a series of hydroxyl radicals, aggravates oxidative stress, propagates lipid peroxidation, and damages neurons, retinal endothelial cells (REC) and retinal pigment epithelium (RPEC), which may catalyze the induction of ferroptosis, and lead to diabetic retinopathy (DR). Renal damage in patients with diabetes nephropathy leads to abnormal iron metabolism, including decreased absorption, increased release and decreased utilization of iron, which aggravates iron deficiency and leads to anemia.

Iron metabolism disorders are highly relevant to a variety of diabetic complications. In diabetic retinopathy (DR), retinal damage has a close connection with oxidative stress, in which iron overload is not only a catalyst but also a cofactor to coordinate oxidative upheaval. Retinal iron content accelerates with age, and diabetes exacerbates pathological iron deposition in the retinal domain (39). The iron influx in the retina, through the Fenton/Haber-Weiss reaction, induces the generation of hydroxyl radicals, aggravates oxidative stress, disseminates lipid peroxidation, and causes damage to neurons, retinal endothelial cells (REC) and retinal pigment epithelial cells (RPEC), which may catalyze the induction of ferroptosis (40). Iron deficiency plays a vital role in chronic kidney disease (CKD). Iron is a component of red cell hemoglobin, and iron deficiency can lead to decreased red cell production and aggravate anemia. Patients with CKD are often accompanied by chronic inflammation and kidney damage. These pathophysiological processes lead to abnormal iron metabolism, including decreased absorption, increased release, and decreased utilization of iron, which aggravates iron deficiency (41). According to experiments, the prevalence of iron deficiency anemia is very high among patients with T2DM, and most patients with diabetes also show iron deficiency (42). Chronic kidney disease (CKD) is often accompanied by anemia, and the mechanism by which iron deficiency leads to impaired erythropoiesis in the context of decreased renal function is remarkable. This may be due to a true or relative deficiency of iron stores, which are absolute and functional iron deficiencies, respectively (41, 43). In addition, the present data strongly suggest that peripheral blood iron levels are related to coronary atherosclerosis, and iron concentration is significantly reduced in acute myocardial infarction (AMI) and coronary artery disease (SCAD) (44). Iron also plays a crucial role in cardiac mitochondrial and cardiomyocyte function. Because iron deficiency may severely perturb mitochondrial energy, and iron overload may react with iron in mitochondria via ROS to generate harmful hydroxyl radicals (45), resulting in the opening of the mitochondrial permeability overpore (46) and the mitochondrial membrane potential depolarization and eventually mitochondrial swelling (47). Mitochondrial dysregulation is associated with various pathological conditions of the heart, ranging from advanced heart failure (caused by iron shortage) to cardiac ischemia (due to iron overload) (48). These deleterious effects are partly due to the increase in oxidative stress. (the mechanism is shown in **Figure 2**).

### 3.4 Application of iron chelating agent in T2DM treatment

Iron chelators are a class of drugs used to treat conditions of iron overload or iron toxicity. By binding to free iron in the body, these drugs form stable complexes, thereby reducing the concentration of free iron and preventing its damage to cells and tissues. The main effects of iron chelators include promoting the excretion of excess iron from the body, reducing iron absorption, and blocking iron recycling in the body (49). Common iron chelators include deferoxamine and aminoglycolic acid, which play an essential role in the treatment of iron-related diseases, such as β-thalassemia major and iron poisoning. Iron chelation therapy can decrease the incidence of diabetes, and strengthening iron chelation and the appropriate combination of chelating agents can reduce insulin resistance (IR) and improve glucose tolerance (50). Iron chelators improve β-cell function and insulin sensitivity by reducing the concentration of free intracellular iron. Firstly, iron chelators can reduce the level of intracellular free iron and reduce the degree of oxidative stress, thereby protecting the structure and function of islet beta cells and helping to maintain the normal secretion of insulin. Secondly, iron chelators can reduce the presence of free iron, reduce the release of inflammatory factors, and alleviate the inflammatory response, which is beneficial to the protection of β cells. In addition, iron chelators can reduce the interference of intracellular iron overload on the insulin signaling pathway, which helps to improve insulin signaling and improve insulin sensitivity (51).

## 4 Copper ion metabolism

### 4.1 Overview of copper ion metabolism

As a component or essential cofactor of many enzymes, copper is involved in numerous physiological pathways, including preventing ROS damage (such as Cu, Zn-SOD), participating in the mitochondrial respiratory chain (such as cytochrome C oxidase), and iron metabolism (such as ceruloplasmin) (52). Precise regulation of copper is achieved by a protein known as a metal chaperone. Copper enters cells through cell surface transport proteins (CTRs), and upon entry, it binds to molecular chaperones and is transported to the Golgi, apo-SOD1 or to the mitochondria. After arriving at the destination, copper is transported to the copper transport P-type ATPase molecules on the Golgi apparatus, including Wilson’s disease protein (ATP7B) and Menkes disease protein (ATP7A), and released. Among them, ATP7B is mainly present in the liver, and Wilson’s disease can damage this protein, leading to the accumulation of Cu. ATP7A is present in extrahepatic tissues, which can be damaged by Menkes disease and hinder the absorption of Cu in the intestinal mucosa, leading to systemic copper deficiency (53). Copper ion homeostasis must be tightly controlled. Copper imbalances can lead to a wide variety of diseases, chief among them are those caused by copper overload. Copper overload includes excessive copper intake and hereditary copper overload. Excessive copper intake is mostly acute poisoning, which is related to copper pollution in tableware and eating foods with excessive copper content. Chronic poisoning has a low incidence and is likely to present a genetic predisposition. Studies have shown that it is usually autosomal recessive transmission, and the genetic origin is not yet clear (54). Hereditary copper overload is further divided into copper-associated infantile cirrhosis and Wilson’s disease.

### 4.2 Wilson’s Disease and diabetes

Wilson’s Disease (WD), also referred to as Hepatolenticular Degeneration (HLD), is an autosomal recessive copper metabolism disorder. WD is caused by a mutation in the copper-transporting ATPase(ATP 7B), which is in charge of copper biliary excretion, resulting in a decrease in the transport of copper from the liver to the bile, and the deposition of excess copper in the liver leading to hepatocellular damage. Copper is released into the blood and deposited in extrahepatic tissues such as the kidneys, cornea, and brain, resulting in clinical symptoms in the corresponding organs (18).

#### 4.2.1 Copper deposition in Wilson’s Disease leads to oxidative stress

Under physiological conditions, the body has an antioxidant defense system that clears oxidative products such as ROS, and oxidative stress refers to the disruption of this balance, damaging tissues and biological macromolecules. Due to the copper absorption process (conversion between the Cu^+^ and Cu^2+)^, positive ions can be released to generate reactive oxygen species (ROS) (55). Oxidative stress can result in peripheral insulin resistance and the impairment of islet β-cell function, which can trigger diabetes and even serious complications (e.g., diabetic cardiovascular disease, diabetic retinopathy, and diabetic neuropathy) (56). Thus, copper deposition due to Wilson’s disease mediates ROS production, leading to diabetes.

#### 4.2.2 Copper deposition in Wilson’s Disease affects zinc

Cu and Zn are a pair of antagonistic microelements. During intestinal absorption, they compete for the common carrier protein, which is metallothionein. Cu increases have an impact on zinc absorption, leading to significant Zn loss from the body, and the reduction of Zn promotes diabetes (see 5) (56).

### 4.3 Disorder of copper metabolism and diabetic complications

Diabetes predisposes to Cu overload, leading to oxidative stress, which ultimately in turn promotes diabetic complications, primarily copper ions in cardiovascular, renal and retinal damage (55). Diabetic complications can be categorized into large and small vascular complications, which are the effects of the chronic hyperglycemic state of diabetes on the body’s vascular system, leading to vascular damage and disease progression. Large vascular complications are diseases of the cardiovascular. Small vessel complications include diabetic neuropathy, diabetic retinopathy and diabetic nephropathy. The association between copper dysmetabolism and diabetic complications is discussed below in terms of large and small vascular classifications.

#### 4.3.1 Disorder of copper metabolism and macrovascular complications

It has been suggested that copper is an antioxidant nutrient with beneficial effects on cardiovascular health. Cu serves as a component of many enzymes, some of which are important for cardiovascular health, such as lysyl oxidase, which is required for the cross-linking of arterial elastin and collagen, and arterial proteoglycan metabolism is also interrupted due to copper deficiency (57). Studies have shown that superoxide dismutase is decreased in patients with myocardial infarction. This is because copper-zinc superoxide dismutase (SOD1) can catalyze the dismutation of superoxide radicals into hydrogen peroxide, which is the key enzyme against ROS. When this enzyme is deficient, cardiomyocytes will be damaged through oxidative stress (58) (**Table 2**).

**Table 2:** Physiological effects of associated copper chaperone proteins and their mediation in cardiac diseases.

Copper-dependent enzymes	Function	Disease consequence
CRT1	Adjust the REDOX state of copper ion; Regulate the homeostasis of copper ions within cells; High-affinity copper transfer protein	Cardiac hypertrophy; Cardiomyopathy accompanied by endocardial fibrosis and cardiac hypertrophy (59)
ATPase	ATP7A: widely expressed, except for the liver in normal conditions; regulate copper transport; regulate ATP hydrolysis rate ATP7B: existed in the liver and certain parts of the mammary tissue, kidney, placenta, and brain; regulate copper transport; regulate ATP hydrolysis rate	High prevalence of congenital heart disease
CCO	Catalyzes the final stage of respiration in cells; Electron transfer protein	Lactic acidosis; Hypertrophic cardiomyopathy
MT	Decrease oxidative stress, and apoptosis; Prevent mitochondrial morphological deterioration and reduction in creatine phosphokinase levels; Intracellular copper scavengers	Cardiac dysfunction and fibrosis (60)
SOD1	Restrain oxidative stress, autophagy and apoptosis; Catalyzes the disproportionation of superoxide to hydrogen peroxide and molecular oxygen; Oxidoreductase	Cardiac injury (inflammation and apoptosis); Early onset cardiac hypertrophy
SOD3	Decreases myocardial inflammation, fibrosis, and apoptosis	HF; Myocardial infarction; Fibrosis and IHD; Left ventricular dilation; Cardiac hypertrophy (61, 62)
CP	Negatively associated with NO; The main Cu carrier in serum; Catalyzes; Converts NO to nitrite *in vivo*; An oxidase for NO	Mortality; IHD; Atherosclerosis; Dyslipidemia; Obesity; DM (63)
LOX	Required for crosslinking of elastin and collagen; Converts lysine into aminoadipic semialdehyde; Oxidase	Concentric cardiac hypertrophy; Systolic dysfunction; Myocardial fibrosis (64)

#### 4.3.2 Disorder of copper metabolism and microvascular complications

Studies have shown that Cu is involved with the EDRF-dependent arteriolar diastolic defects and impaired peripheral blood flow that occur in diabetes, leading to reduced intraneural blood flow and impaired peripheral nerve function. EDRF is thought to be a thiol adduct of nitric oxide or nitric oxide. In diabetic patients, the internal elastic layer of the arteries accumulates glycated proteins, which are able to bind Cu, and the abundance of Cu within the arterial wall will enhance hyperglycemia through metal catalysis, leading to ROS production via glucose autoxidation, disrupting NO or NO adducts, preventing normal EDRF-mediated diastole, and resulting in defective arterial relaxation and reduced peripheral blood flow (65). In addition to this, copper deficiency in the body may also lead to small vessel complications depending on the role of SOD. Superoxide dismutase (SOD), which is mammals’ main antioxidant defense system, requires the activation of metal catalysts (Cu or Mn). Whereas, in the presence of Cu deficiency, the ability of SOD to protect NO^-^mediated vasodilation is greatly reduced (66).

In addition, increased production of reactive oxygen species may itself impair EDRF-dependent vasodilation. Patients with diabetes have excessively high levels of reactive and exchangeable copper in their tissues, thus causing a variety of small vessel complications associated with peripheral blood reduction (65).

**Figure 3:**
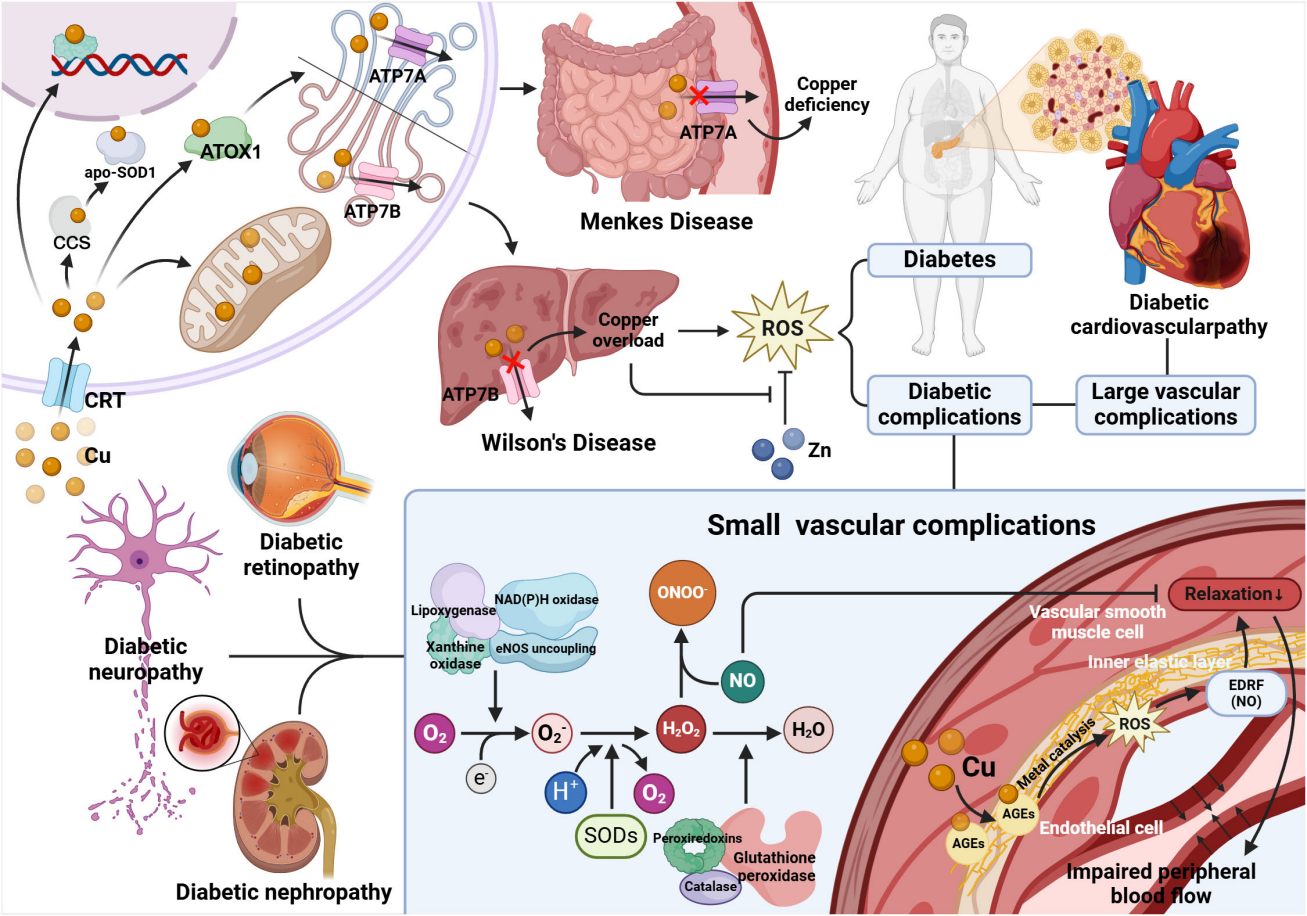
Relationship between copper metabolism disorder and diabetes and its complications. Wilson's disease protein (ATP7B) is mainly present in the liver, and Wilson's disease can damage this protein, leading to the accumulation of Cu. ATP7A is present in extrahepatic tissues, which can be damaged by Menkes disease and hinder the absorption of Cu in the intestinal mucosa, leading to systemic copper deficiency. Copper overload will lead to diabetes and diabetes complications through oxidative stress, in which diabetes small vessel disease is closely related to EDRF-dependent arterial relaxation. In diabetic patients, the internal elastic layer of the arteries (composed of elastin) accumulates glycated proteins that bind Cu, and the Cu enriched in the arterial wall catalyzes the destruction of NO or NO adducts, which is EDRF, by the metal catalysis, which prevents normal EDRF-mediated diastole, leading to arterial diastolic defects and impaired peripheral blood flow. And SOD can protect against NO^-^mediated vasodilation. Superoxide (O^2-^) is produced by mitochondrial enzymes, lipoxygenase, nitric oxide synthase (NOS), xanthine oxidase and NADPH oxidase. Superoxide dismutase (SOD) converts superoxide to H_2_O_2_. Then, peroxide reductase (Prx), glutathione peroxidase (GPx), and catalase reduce H_2_O_2_ to water. Nitric oxide (NO) is rapidly deactivated through reacting with O^2-^, resulting in the generation of the peroxynitrite (ONOO^-^), which is a strong oxidant. Therefore, SOD is the first line of defense against the toxicity of superoxide anion radicals. Moreover, this enzyme protects NO and its ability to mediate vasodilation.

Diabetes leads to pathogenic copper overload, accompanied by enhanced copper-mediated oxidative stress, which in turn results in tissue damage in the nerves, retina, kidneys, heart and blood vessels, known as diabetic complications. For example, Renal tubular dysfunction in diabetes is likely a consequence of copper overload in the kidneys, in which copper-induced oxidative stress impairs EDRF-dependent vasodilation, resulting in reduced renal blood flow, which is the key to the molecular mechanism that causes diabetic nephropathy (67). (The above mechanism is shown in **Figure 3**).

## 5 Zinc ion metabolism

### 5.1 Zinc deficiency is associated with type 1 diabetes, and zinc supplementation can improve insulin sensitivity

Insufficient insulin production is the sole cause of T1DM. Zinc plays a variety of roles in the insulin signaling pathways, pancreatic storage and secretion, and structure and conformation of insulin (68). Therefore, zinc deficiency may contribute to the development of diabetes. According to the model study, the risk of diabetes will be significantly increased in drinking groundwater with low zinc content, and there is a close relationship between zinc and diabetes (69, 70). In most mammals, insulin is stored as zinc crystals in β cells of the pancreas (71). Moreover, adding zinc to insulin leads to a conformational change and strengthens the binding of insulin to its receptor (72). According to a series of animal experiments, zinc deficient rats show peripheral insulin resistance, and zinc supplementation improves insulin sensitivity to a certain extent (73).

### 5.2 Protective effect of zinc on diabetic complications

It is well known that the beneficial impacts of zinc supplementation in diabetes include modulating oxidative stress, promoting glycemic control, and reducing insulin resistance. At the same time, zinc supplementation also has a beneficial effect on the secondary complications of diabetes. First, zinc supplementation may decrease the risk of dyslipidemia and cardiovascular disease associated with diabetes. A number of clinical studies have shown that zinc supplementation has significant benefits on blood lipids. Zinc ions can promote the synthesis and stability of HDL, increase its functional activity, and thus increase the level of HDL in the body. In addition, zinc ions can regulate the synthesis and metabolism of LDL and reduce its level in the blood, thereby reducing the risk of atherosclerosis. Zinc ions are involved in regulating the synthesis and metabolism of cholesterol in the body and help maintain the balance of cholesterol (74). It is able to reduce the levels of LDL cholesterol and total cholesterol while increasing the level of HDL cholesterol, thereby improving the lipid profile and reducing the risk of cardiovascular disease (75, 76). Second, zinc supplementation slowed the progression of diabetic nephropathy. Clinical trials have shown that zinc supplementation is beneficial in reducing urinary protein excretion and inflammation in patients with diabetic nephropathy (77, 78). According to another animal experiment, zinc deficiency leads to pathological changes in the renal cortex of rats, including enlargement of Bowman’s space, enlargement of mitochondria in the proximal distal flexural tubules, and fibrosis of the cortical interstitium, while zinc supplementation can minimize histopathological changes (79). In addition, zinc supplementation can improve diabetic cataract by regulating lens protein and polyol pathway, and can also reduce diabetic peripheral neuropathy (80).

## 6 Calcium ion metabolism

Calcium is essential for bone health and many important physiologic functions. Most of the calcium present in the body is in the form of Ca^2+^ ions, complexes, and inorganic salts (52). Calcium ions play a key role in pancreatic islet cells and are involved in insulin secretion.

### 6.1 Role of calcium ions in insulin secretion from pancreatic β cells

In islet beta cells, glucose-induced rapid increase of Ca^2+^ is a central signaling messenger that triggers insulin secretion (81). Insulin secreted by pancreatic β cells is stored in large dense core vesicles (LDCV), which are recruited to the plasma membrane and secreted insulin by regulating exocytosis. In β-cells, Glucose metabolism in β-cells stimulates mitochondria to produce ATP, which causes ATP-sensitive K^+^ channel (KATP) membranes to depolarize closure and voltage-dependent Ca^2+^ channels to open, promote Ca^2+^ influx, and bind cations to gelsolin and synaptotagmin, which are conducive to cortical actin (limiting but not preventing vesicles from entering the plasma membrane) and membrane fusion, respectively (82).

### 6.2 Disturbed calcium homeostasis in diabetes

#### 6.2.1 Diabetes and hypocalcemia

Diabetic patients have impaired renal function due to hypovolemia. In the case of renal failure, kidney’s phosphorus excreted is reduced, resulting in hyperphosphatemia. Hyperphosphatemia triggers hypocalcemia through interfering with the kidney’s phosphorus excretion (83). Diabetes also has hypomagnesemia, which leads to hypocalcemia. Hypomagnesemia in diabetic patients may be caused by poor oral intake, gastrointestinal malabsorption, and increased renal magnesium excretion. Mg^2+^ depletion impairs parathyroid hormone (PTH) secretion and renal tubules and bone are resistant to PTH action, resulting in hypocalcemia (83, 84). In addition, in patients with type 1 diabetes, a small decrease in PTH secretion will also reduce blood calcium levels (83).

#### 6.2.2 Diabetes and hypercalcemia

In type 2 diabetes, reduced glucose transport through normal insulin stimulation leads to increased intracellular free calcium concentration, increasing the demand for insulin, resulting in excessive insulin production and secretion, leading to insulin resistance mediated by hyperparathyroidism. In addition, patients with diabetes who take thiazide diuretics are more likely to develop hypercalcemia (85).

### 6.3 Calcium signaling pathway as a therapeutic target in diabetes

As mentioned above, insulin secretion depends on calcium signaling channels, and calcium deficiency can lead to insulin resistance and decreased insulin secretion. Therefore, regulating calcium channels can improve insulin resistance and increase insulin secretion.

Polymorphisms in the calmodulin-dependent kinase subtype D (CaMK1D) gene region are linked to an increased incidence of diabetes (86). However, in a European population-based study, no significant correlation between T2D and CaMK1D SNP was identified. Therefore, the function of CaMK1D gene in the pathophysiology of T2D is still ambiguous (87). A study using mice as a model showed that highly selective calmodulin-dependent kinase inhibitors, such as CaMK1 selective inhibitors, can restore insulin sensitivity, confirming CaMK1D as a target for diabetes treatment (86).

## 7 Metal metabolism as a new direction in diabetes research and treatment

### 7.1 Antioxidants

The effect of metal ions on diabetes and its complications, mainly through oxidative stress, promotes the use of antioxidant compounds in the treatment of diabetes. Antioxidant therapy includes low molecular weight tissue-permeable compounds such as vitamin E, lipoic acid, or acetylcysteine (88). In addition to common antioxidants, C-peptide, as a natural antioxidant, is secreted together with insulin. Research has shown that C-peptides mainly exhibit antioxidant, anti-apoptotic, and anti-inflammatory effects by binding to cell surface signaling molecules to activate downstream pathways or regulate intracellular transcription processes. It can protect beta cells from oxidative stress and inflammation damage, and also has a protective effect on other cell types (89, 90). Therefore, C-peptide has potential therapeutic value in the prevention and treatment of diabetes and its complications (89).

In addition, the role of alpha-lipoic acid (ALA) as a powerful antioxidant in the treatment of diabetes has been studied. Alpha-lipoic acid and its reduced form, DHLA, are considered to be powerful natural antioxidants with the ability to scavenge many reactive oxygen species, which can inhibit oxidative stress-induced pancreatic β Cell damage has a potential protective effect. Antioxidants including ala can improve endothelial nitric oxide synthase (eNOS) to form NO, but not O^2-^, to improve vascular elasticity, which holds significant importance in the treatment of diabetic complications (91, 92).

### 7.2 Metal chelates

Essential metals are critical in maintaining intracellular homeostasis, but overloading may increase the production of reactive oxygen species (ROS), thereby leading to metal-induced toxicity. Iron and copper are the two metals with the greatest potential for ROS production. They can both be chelated by ethylenediaminetetraacetate (93). Disodium ethylenediaminetetraacetate is a synthetic amino acid that has a high affinity for ions with +2 ~ +6 valence. It is able to trap metal ions and excrete them through the kidneys or bile, thus removing the metal from the body (94). Patients with chronic renal failure and ischemic heart disease were treated with ethylenediaminetetraacetic acid (EDTA) chelation therapy alone to promote the elimination of copper/iron ions from the body and reduce the damage to kidney cells and cardiomyocytes (95).

In addition, the transition metal chelator Trientine exerted a significant beneficial effect on endothelium-dependent diastole mediated by EDHF and NO in mesenteric vessels. The effects of Trientine, which are attributed to protection against the harmful effects of oxidative stress, may imply potential therapeutic approaches for diabetic microangiopathy and vasculopathy (96). It has been shown that diabetic patients show structural improvement in the heart after long-term Trientine treatment (97).

### 7.3 Classical hypoglycemic agents

Metal ions also play a role in medications commonly employed in the treatment of diabetes, including angiotensin II receptor blocker (ARB), angiotensin-converting enzyme inhibitors (ACEIs), and metformin (98). ACEIs and ARB inhibit metal-catalyzed oxidation of ascorbic acid *in vitro* and reduce AGE formation. Metformin can play a role in AMP-activated protein kinase by interacting with mitochondrial copper, trapping α-dicarbonyl groups and leading to reduced gluconeogenesis (94).

### 7.4 Proprietary Chinese medicines

There are many reports regarding the benefits of plant nutrients and extracts (e.g., resveratrol, quercetin, polyphenols, rutin) against diabetic complications. Among these compounds, some can inhibit the formation of AGE in diabetic animals. Part of their action mechanism might be related to limiting the absorption of metal ions or facilitating their excretion through chelating activity (99). For example, rutin can inhibit the small intestine’s absorption of glucose, as well as stimulate β Cells to secrete insulin and increase glucose uptake by tissues, thereby lowering blood glucose. In addition, rutin can reduce the formation of inflammatory cytokines by inhibiting the formation of age, as well as inhibit aldose reductase and reduce the concentration of sorbitol in erythrocytes, maintaining intracellular NADPH levels, and reduce oxidative stress, thereby protecting against dyslipidemia and hyperglycemia induced cardiovascular disease, liver injury, neuropathy, and nephropathy (100).

### 7.5 Metallothionein

Metallothionein (MT) is a metal-binding protein that can be induced by numerous conditions, including zinc supplementation. It prevents or treats diabetes and its complications through anti-inflammatory effects, anti-apoptotic, and anti-OS, as well as reducing the acute toxic effects of heavy metals. Some studies have demonstrated successful treatment and prevention of type 2 diabetes, diabetic neuropathy, diabetic nephropathy, and diabetic complications using MT protein therapy. In addition, MT prevents diabetes-induced myocardial ER stress, thus contributing to the prevention and treatment of diabetic cardioprotection (101) (**Table 3**).

**Table 3:** Metal related drugs and treatment of diabetes.

Classification	Drugs	Function
Antioxidants	Vitamin E, Lipoic acid, or Acetylcysteine	Clear reactive oxygen species and reduce the impact of oxidative stress caused by metal ions
Metal chelates	Ethylenediaminetetraacetate, Trientine	Chelate with metal and reduce metal-induced toxicity
Classical hypoglycemic agents	ACEIs, ARB	Inhibit metal-catalyzed oxidation of ascorbic acid *in vitro* and reduce AGE formation
	Metformin	Trap α-dicarbonyl groups and lead to reduced gluconeogenesis
Proprietary Chinese Medicines	e.g., rutin, polyphenols, quercetin, resveratrol	Inhibit AGE formation, Limit the uptake of metal ions or Promote their excretion through chelating activity
Metallothionein		Anti-inflammatory effects, Anti-apoptotic, Anti-OS, or Reduce the acute toxic impacts of heavy metals

## 8 Conclusion

In summary, the involvement of metallic ions in the etiology and progression of diabetes and its associated complications is multifaceted and pivotal. Dysregulations in the metabolism of minerals such as iron, copper, and zinc not only impact insulin secretion and cellular integrity but are also integrally linked to the onset and exacerbation of diabetic complications. Despite the promising therapeutic implications of iron-chelating agents and antioxidants in managing diabetes, comprehensive investigative efforts and empirical clinical validation are imperative. Future endeavors should aim at a profound comprehension and precise modulation of these processes.
